# Validating Candidate Congenital Heart Disease Genes in *Drosophila*

**DOI:** 10.21769/BioProtoc.2350

**Published:** 2017-06-20

**Authors:** Jun-yi Zhu, Yulong Fu, Adam Richman, Zhe Han

**Affiliations:** 1 Center for Cancer and Immunology Research, Children’s National Medical Center, 111 Michigan Ave. NW, Washington, DC, USA; 2 Department of Pediatrics, The George Washington University School of Medicine and Health Sciences, Washington, DC, USA

**Keywords:** *Drosophila*, High-throughput screening, Congenital heart disease, Lethal rate, Heart morphology

## Abstract

Genomic sequencing efforts can implicate large numbers of genes and *de novo* mutations as potential disease risk factors. A high throughput *in vivo* model system to validate candidate gene association with pathology is therefore useful. We present such a system employing *Drosophila* to validate candidate congenital heart disease (CHD) genes. The protocols exploit comprehensive libraries of UAS-GeneX-RNAi fly strains that when crossed into a 4×Hand-Gal4 genetic background afford highly efficient cardiac-specific knockdown of endogenous fly orthologs of human genes. A panel of quantitative assays evaluates phenotypic severity across multiple cardiac parameters. These include developmental lethality, larva and adult heart morphology, and adult longevity. These protocols were recently used to evaluate more than 100 candidate CHD genes implicated by patient whole-exome sequencing (Zhu *et al*., 2017).

## Background

The use of the *Drosophila* model to elucidate molecular mechanisms underlying human diseases is well documented (Bier and Bodmer, 2004; Cagan, 2011; Zhang *et al*., 2013; Owusu-Ansah and Perrimon, 2014; Diop and Bodmer, 2015; Na *et al*., 2015), and 75% of human disease associated genes are represented by functional homologs in the fly genome (Reiter *et al*., 2001). While it is a challenge to link *Drosophila* developmental phenotypes directly to patient symptoms, *Drosophila* can be used as a very sophisticated and efficient platform to test and validate candidate disease gene function in development, and this can readily be scaled to evaluate large number of candidate genes identified from patient genomic sequencing efforts. *Drosophila* has been used to study genes related to CHD for over 20 years, based on evolutionarily conserved genetic mechanisms of heart development (Bier and Bodmer, 2004; Olson, 2006; Yi *et al*., 2006). We developed a highly efficient cardiac-targeted gene silencing approach in flies to examine effects on heart structure and function for fly homologs of candidate CHD genes (Zhu *et al*., 2017).

## Materials and Reagents

1. 1,250 µl pipette tips (BioExpress, GeneMate, catalog number: P-1234-1250)
2. 200 µl pipette tips (BioExpress, GeneMate, catalog number: P-1237-200)
3. 10 µl pipette tips (BioExpress, GeneMate, catalog number: P-1234-10XL)
4. Permanent marker
5. Fisherbrand™ plastic Petri dishes (Fisher Scientific, catalog number: S33580A)
6. Microscope slides (VWR, catalog number: 16004-430)
7. 24 × 50 mm gold Seal™ cover slips (Thermo Fisher Scientific, Thermo Scientific™, catalog number: 3322)
8. Heart-specific Gal4 driver line, 4×Hand-Gal4/Cyo (Generated by Dr. Zhe Han)
9. UAS RNAi transgenic strains targeting *Drosophila* orthologs of candidate CHD genes (Bloomington *Drosophila* Stock Center)
10. Carbon dioxide (Roberts Oxygen Company)
11. Fly food (Meidi Laboratories)
12. Vaseline (COVIDIEN™)
13. Schneider’s *Drosophila* medium (Thermo Fisher Scientific, Gibco™, catalog number: 21720001)
14. Paraformaldehyde solution, 4% in PBS (Alfa Aesar, Affymetrix/USB, catalog number: J19943)
15. Phosphate buffered saline (PBS), prepared from 10× PBS, pH 7.4 (GENAXY, catalog number: 40-029)
16. Bovine serum albumin, Powder (Santa Cruz Biotechnology, catalog number: sc-2323)
17. Triton X-100 (Fisher Scientific, catalog number: BP151-100)
18. Alexa Fluor 555 Phalloidin (Thermo Fisher Scientific, Invitrogen™, catalog number: A34055)
19. EC11 anti-Pericardin primary anti-mouse antibody (Developmental Studies Hybridoma Bank, catalog number: EC11)
20. Biotin-conjugated goat anti-mouse antibody (Vector Laboratories, catalog number: SP-1100)
21. Streptavidin (Cy5) (Thermo Fisher Scientific, Invitrogen™, catalog number: SA1011)
22. VECTASHIELD antifade mounting medium with DAPI (Vector Laboratories, catalog number: H-1200)
23. Electron microscopy science clear nail polish (Electron Microscopy Science, catalog number: 72180)

## Equipment

1. Gilson P200 pipette classic large plunger (Gilson, model: P200)
2. Vannas spring scissors–3 mm cutting Edge (Fine Science Tools, catalog number: 15000-10)
3. Dumont #5 forceps (Roboz Surgical Instrument, catalog number: RS-4955)
4. Ultimate Flypad (Genesee Scientific, catalog number: 59-172)
5. Stereo microscope (ZEISS, model: Stemi 305)
6. Zeiss ApoTome.2 microscope using a 20× Plan-Apochromat 0.8 N.A/air objective (ZEISS, model: Apotome.2)
7. *Drosophila* incubator set to 25 °C and 29 °C (Panasonic Healthcare, model: MIR-154-PA)

## Software

1. ImageJ software Version 1.49

## Procedure

### A. High-throughput gene function validation system in *Drosophila*

**Figure 1:**
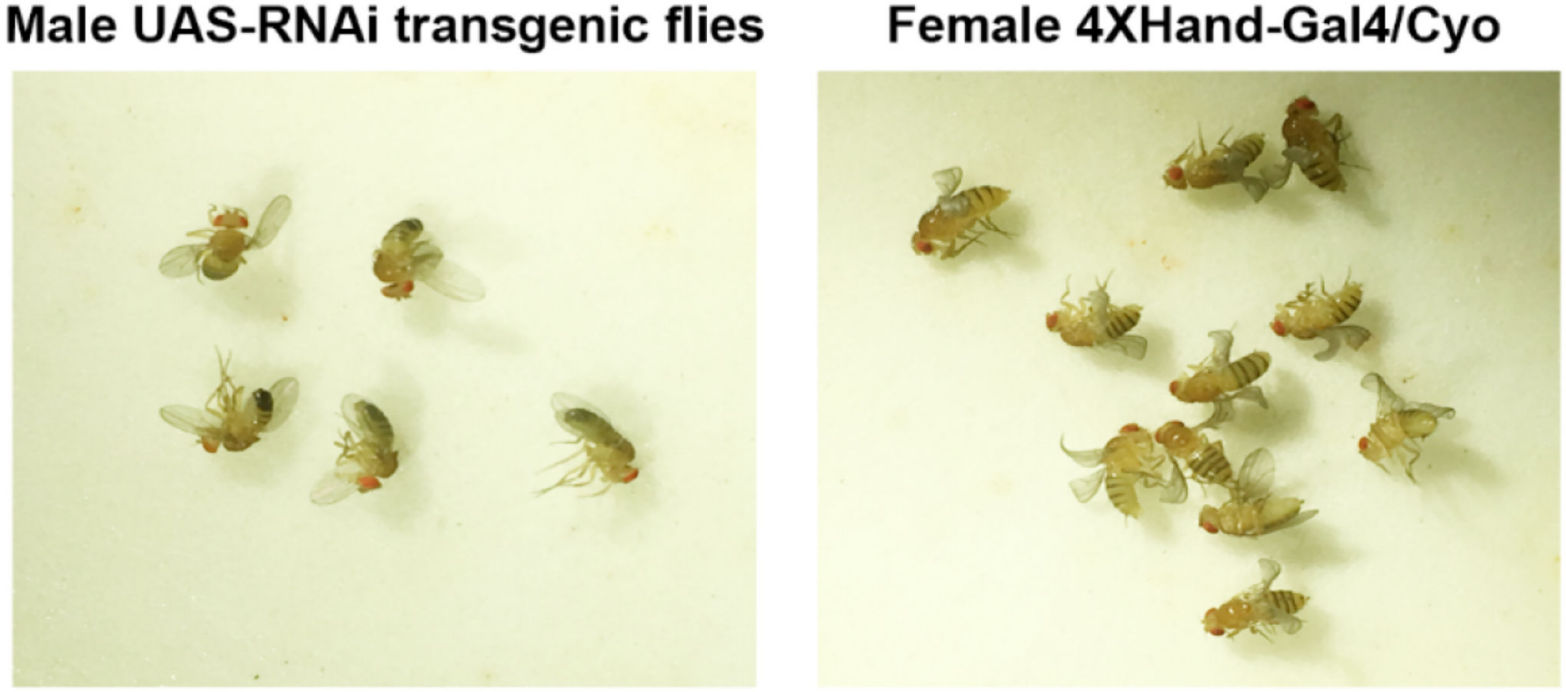
5 male homozygous UAS-RNAi transgenic flies are crossed to 10–15 4-day-old virgin female flies of genotype 4×Hand-Gal4/Cyo

**Figure 2:**
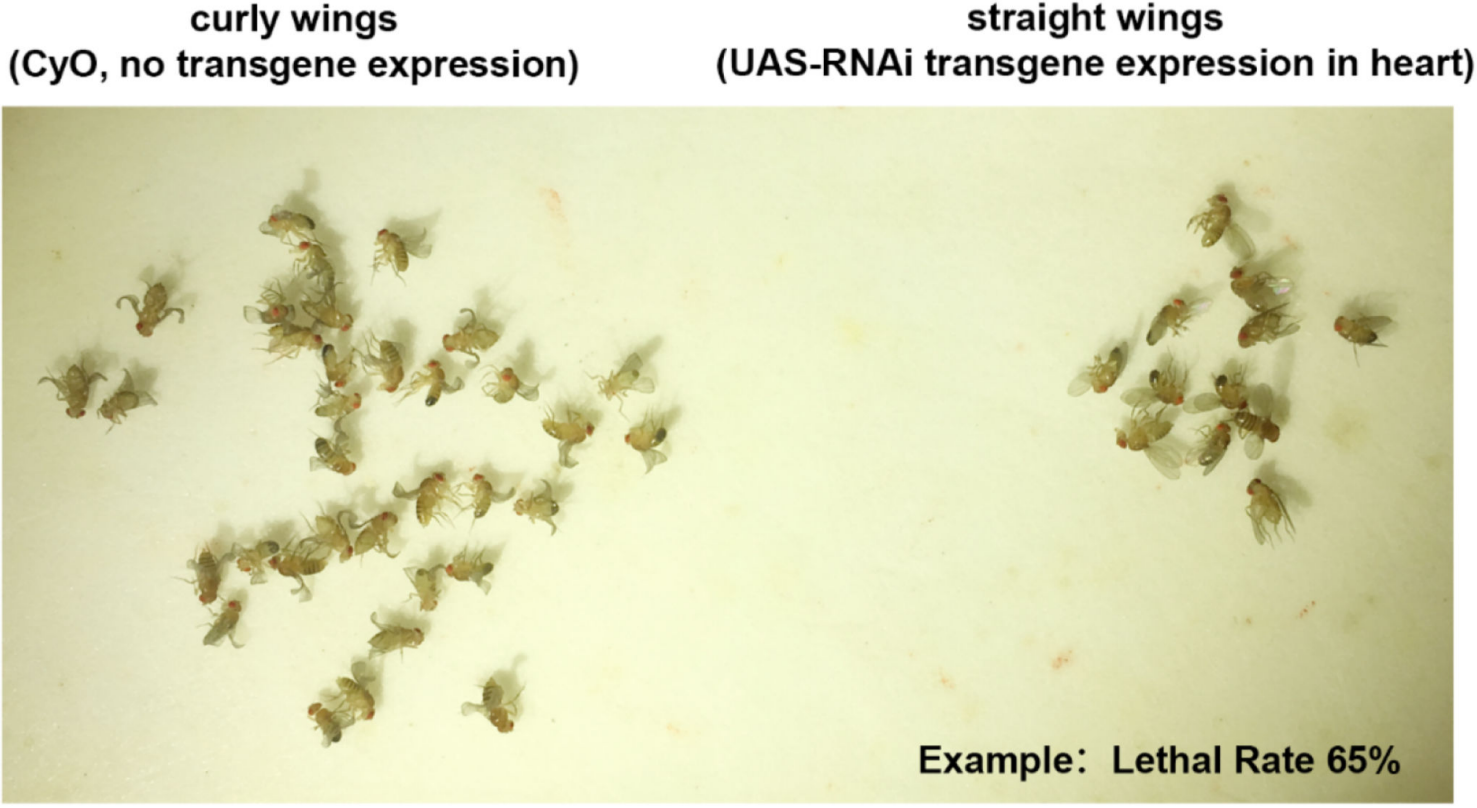
Progeny adult flies emerge and the number of adult flies with curly wings (CyO, no transgene) vs. straight wings (RNAi transgene expressed in cardioblasts) are recorded An example of lethal rate of approx. 65% is shown.

1. 5 male flies homozygous for a UAS-RNAi transgene (targeting the *Drosophila* ortholog of candidate CHD gene) are combined with 10 to 15 4-day-old virgin female flies of genotype 4×Hand-Gal4/Cyo (Figure 1) at 25 °C.
2. The flies are maintained at 25 °C and are transferred daily to a fresh vial of fly food for 5–6 days.
3. Each day the emptied vial containing freshly laid eggs is transferred to 29 °C to boost UAS-transgene expression. At the end of this process 5–6 vials of flies are developing at 29 °C.
4. As adult progeny flies emerge over a period of four to five days they are anesthetized with CO_2_ on a fly pad (Genesee Scientific) and the numbers of curly winged (CyO, no RNAi transgene) vs. straight winged (RNAi transgene expressed in cardioblasts) flies are recorded (Figure 2). Counting continues until at least 200 curly winged flies have been recorded.
5. The [developmental] lethal rate attributable to target gene silencing in the heart is calculated as (Curly - Straight)/Curly × 100% = % Mortality.

### B. Adult survival assay

**Figure 3:**
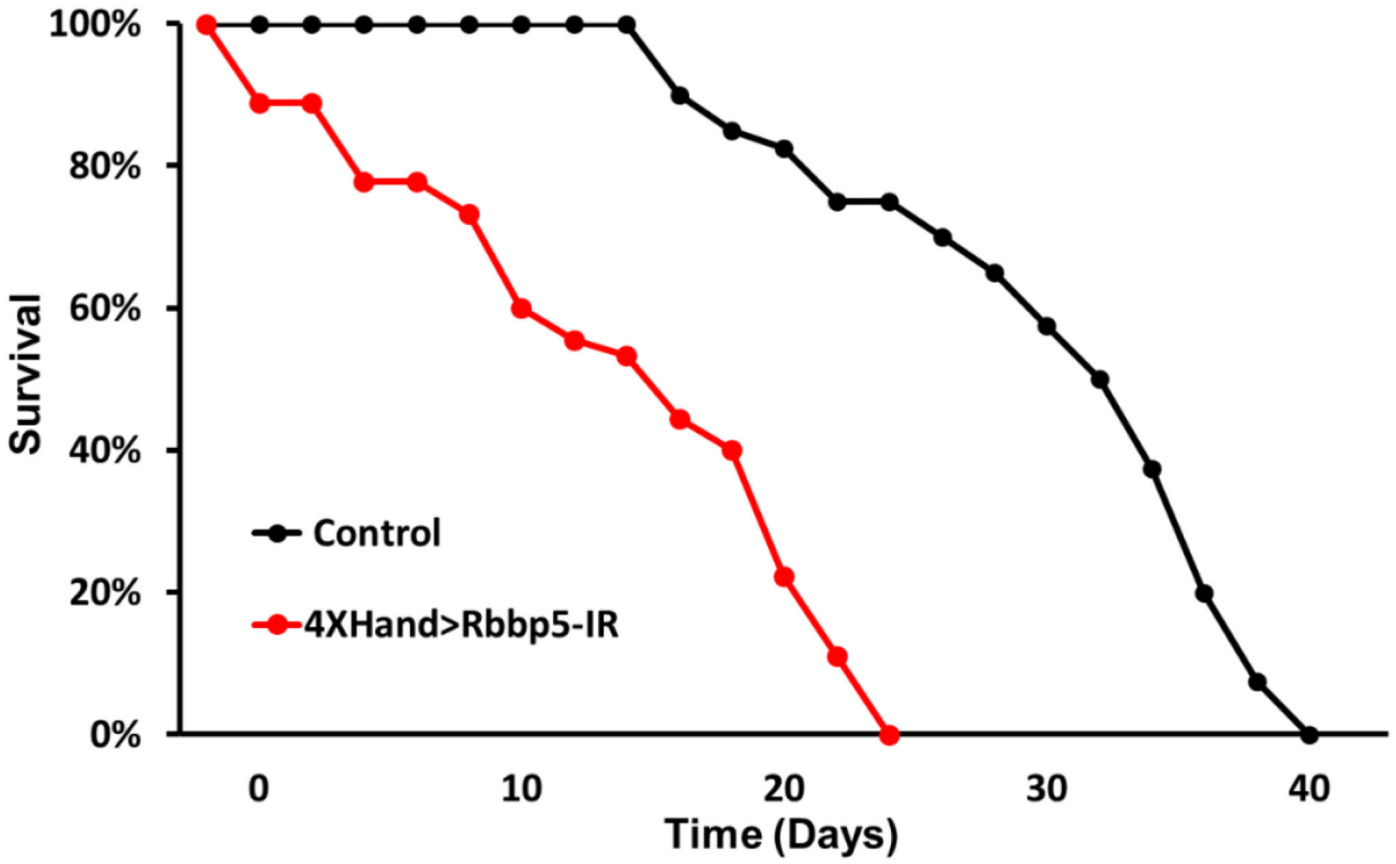
Survival curves for control flies (no RNAi transgene) and flies expressing RNAi targeting the *Rbbp5* gene in cardioblasts Heart-specific *Rbbp5* knockdown significantly reduces longevity.

1. At least 60 adult progeny flies with straight wings (4×Hand-Gal4 driven UAS-RNAi transgene expression in cardioblasts) are collected and maintained at 29 °C. This number of flies can typically be collected in one to two days. Maintain no more than 15 flies per vial. To obtain sufficient numbers of straight winged progeny flies at least 5 crosses should be set up. In the case of RNAi transgenes that induce high mortality, more than 10 crosses should be established.
2. The survival assay initiates immediately upon fly collection. The number of live flies is thereafter recorded every two days until all flies have died.
3. Flies are transferred every two days to a fresh vial of fly food (to prevent flies from becoming stuck in wet food).
4. A survival curve is generated that plots % surviving flies against time (Figure 3).

### C. Adult heart morphology

**Figure 4:**
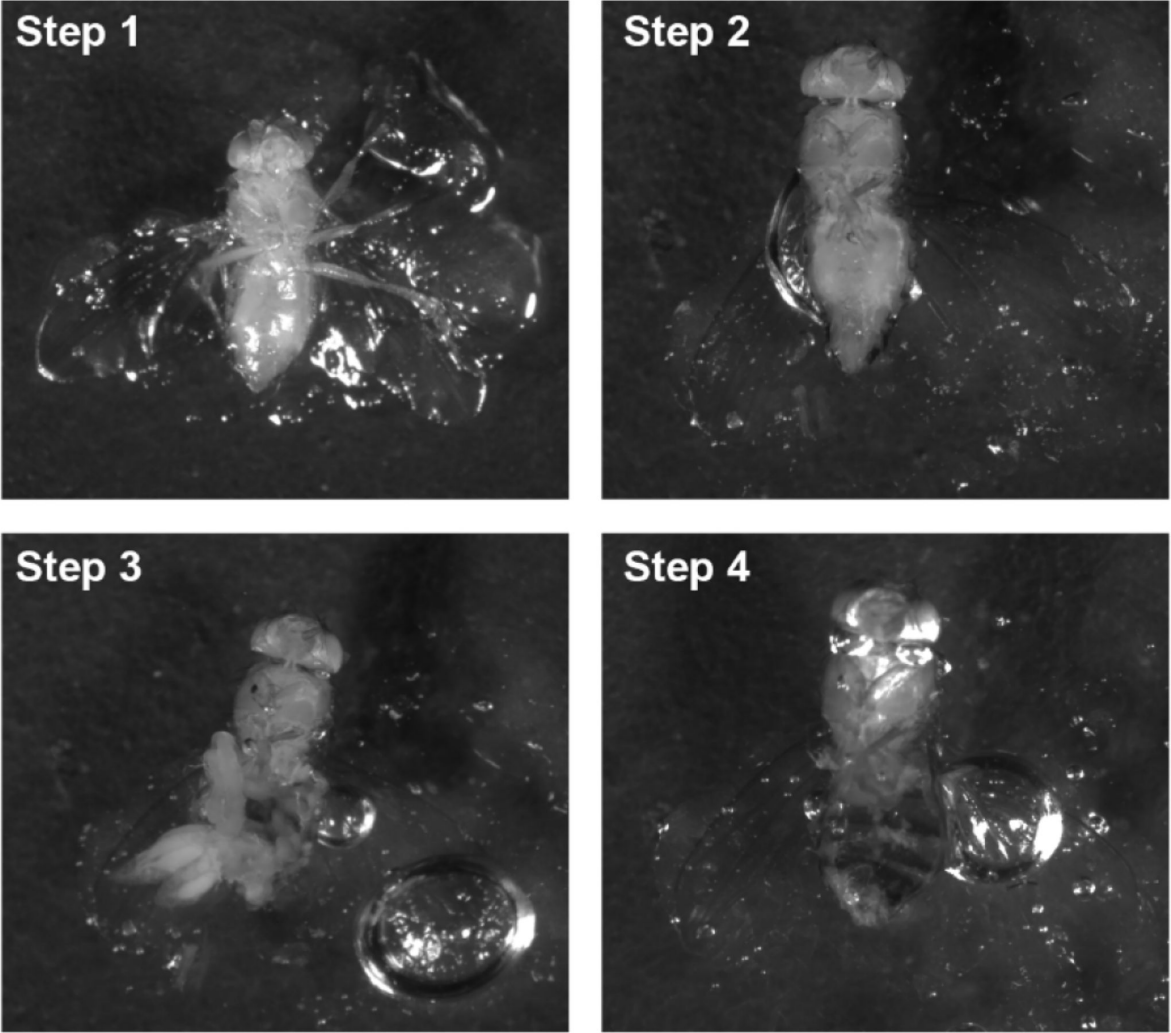
Adult fly dissection Step 1: Stick the fly on the Petri dish; Step 2: Remove the fly legs; Step 3: Open the body from bottom; Step 4: Remove the organs.

**Figure 5:**
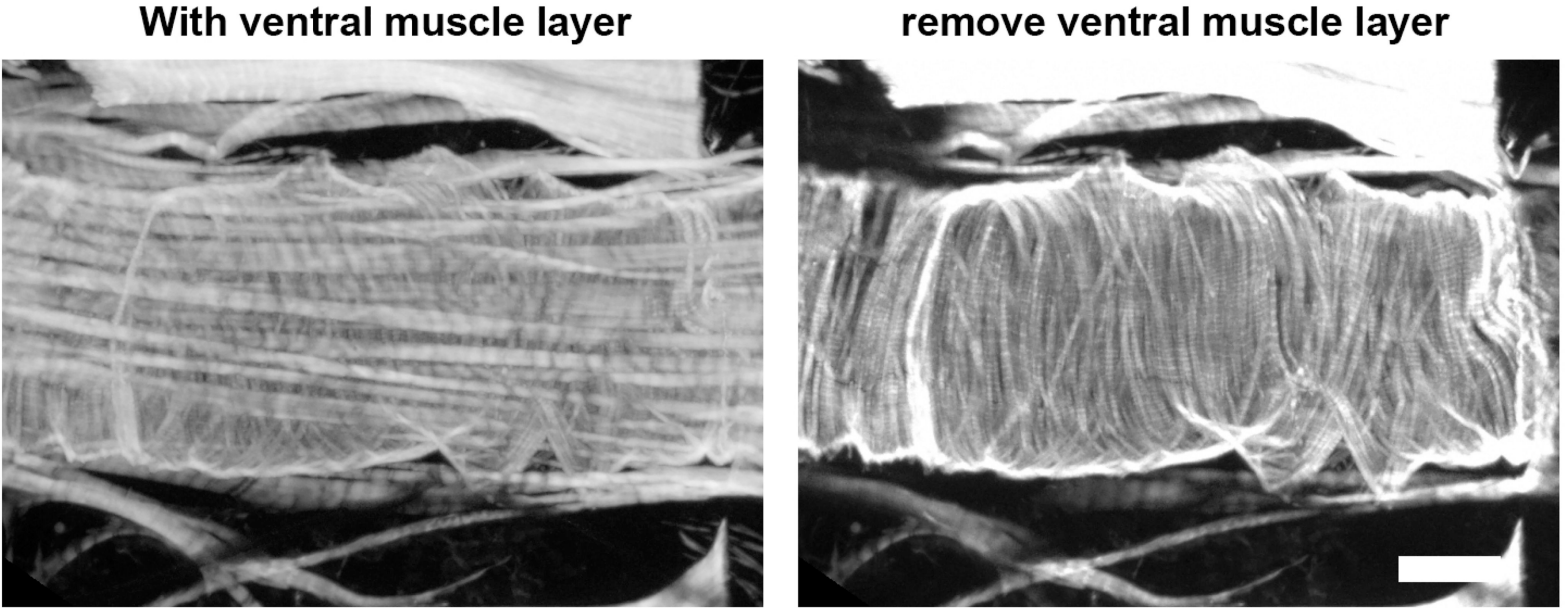
Z-stack layers that exclude ventral muscle fibers are selected for analysis Scare bar = 50 µm.

**Figure 6:**
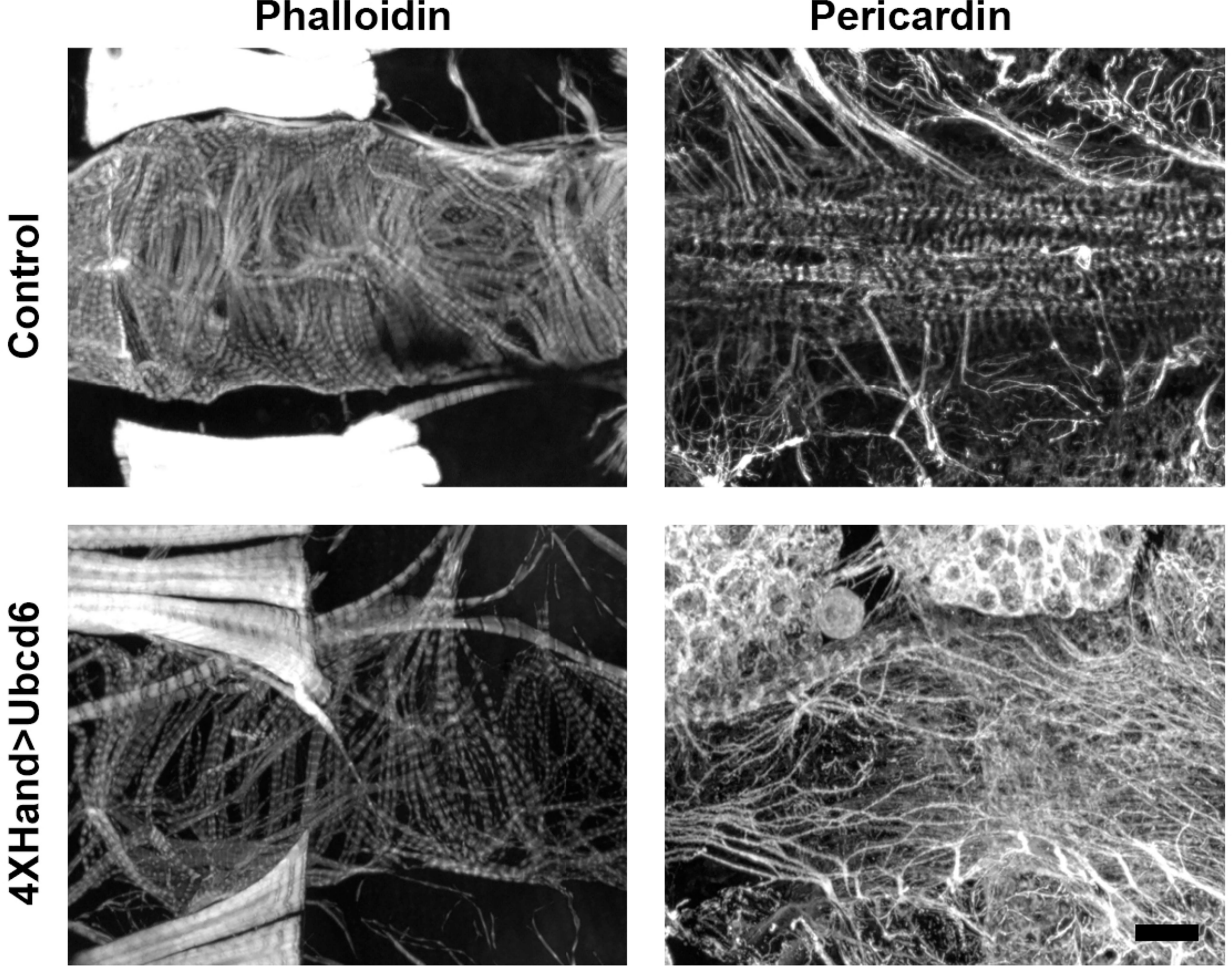
Samples of reduced myofibrillar density and increased pericardin deposition Scale bar = 50 µm.

1. Six to ten straight wing adult progeny flies (RNAi transgene expressed in cardioblasts) and six to ten curly wing control progeny adult flies (no RNAi transgene) are anesthetized with CO_2_ and carefully immobilized (ventral side up) in the bottom of a Petri dish by gently affixing flies in vaseline (Vogler and Ocorr, 2009). Each fly genotype is inscribed directly on the petri dish using a permanent marker. The flies remain in the same Petri dish throughout the procedure until being mounted for microscopic examination. Processing experimental and control flies simultaneously eliminates variability that might result from separate treatments.
2. The legs are removed by amputation using fine spring scissors.
3. Schneider’s *Drosophila* medium (~20 ml per dish) is added by pouring from one side of the Petri dish until flies are completely submerged.
4. Using scissors, begin at the rostral end of the fly and cut circumferentially and continuously to remove the entire ventral abdominal cuticle, revealing the inner organs.
5. Remove the internal organs (viscera) that tend to float free of the abdominal cavity by pipetting adjacent medium up and down 5 to 6 times using a P200 pipette set at 200 µl volume.
6. Remnant viscera and fat body tissue are delicately cleared away using fine forceps (Figure 4).
7. Pour out the original medium. Add fresh Schneider’s *Drosophila* medium (~20 ml) to the Petri dish by pouring from one side until flies are completely submerged to wash the fly carcasses. Pour out the wash medium.
8. Add ~20 ml formaldehyde solution (4% in PBS) to the Petri dish by pouring from one side until flies are completely submerged. Fix for 10 min at room temperature. Pour out the fixative solution.
9. Rinse carcasses by adding ~20 ml PBS to the Petri dish by pouring from one side until flies are completely submerged. Pour out the PBS. Repeat the rinse procedure another 2 ×.
10. Add ~20 ml BSA solution (2% in PBS) containing 0.1% Triton X-100 to the Petri dish by pouring from one side until flies are completely submerged. Incubate for 30 min at room temperature. Pour out the BSA solution.
11. Add ~20 ml PBS containing Alexa Fluor 555 Phalloidin (1:1,000 dilution) and anti-Pericardin (EC11) mouse primary antibody (1:500 dilution) to the Petri dish by pouring from one side until flies are completely submerged. Incubate overnight at 4 °C in the dark.
12. Remove Petri dish from 4 °C.
  Note: The following steps need not be performed in the dark.
13. Rinse 3 × with PBS at room temperature as described in step C9.
14. Add ~20 ml PBS and incubate for 20 min at room temperature. Repeat 3 ×.
15. Add ~20 ml PBS containing Biotin-conjugated goat anti-mouse antibody (1:500 dilution). Incubate for 2 h at room temperature.
16. Rinse 3 × with PBS at room temperature as described in step C9.
17. Add ~20 ml PBS and incubate for 20 min at room temperature. Repeat 3 ×.
18. Add ~20 ml PBS containing Streptavidin Cy5 (1:1,000 dilution) and incubated for 1 h at room temperature.
19. Rinse 3 × with PBS at room temperature as described in step C9.
20. Add ~20 ml PBS and incubate for 20 min at room temperature. Repeat 3 ×.
21. Mount heart tissue on a glass slide in ~2 ml VECTASHIELD antifade mounting medium.
22. Apply a cover slip using forceps.
23. Seal edges of cover slip using nail polish.
24. Confocal imaging is performed using a Zeiss ApoTome.2 microscope fitted with a 20× Plan-Apochromat 0.8 N.A/air objective.
25. Control groups are imaged first to establish light intensity and exposure time. An exposure time is found at which the image is saturated, and then reduced to a set point of approx. 70% saturation to allow comparison of fluorescence intensity across genotypes.
26. The entire heart is imaged by collecting Z-stack images. Same number of samples of each phenotype are imaged.
27. Images are exported to tiff file format.
28. ImageJ software Version 1.49 is used for image processing.
29. Z-stack projections are screened and image levels containing cardiac myofibers are selected for analysis, avoiding the ventral muscle layer that underlies the heart tube (Figure 5).
30. Samples of reduced myofibrillar density and increased pericardin deposition are shown (Figure 6).

### D. 3^rd^ instar larva heart morphology

**Figure 7:**
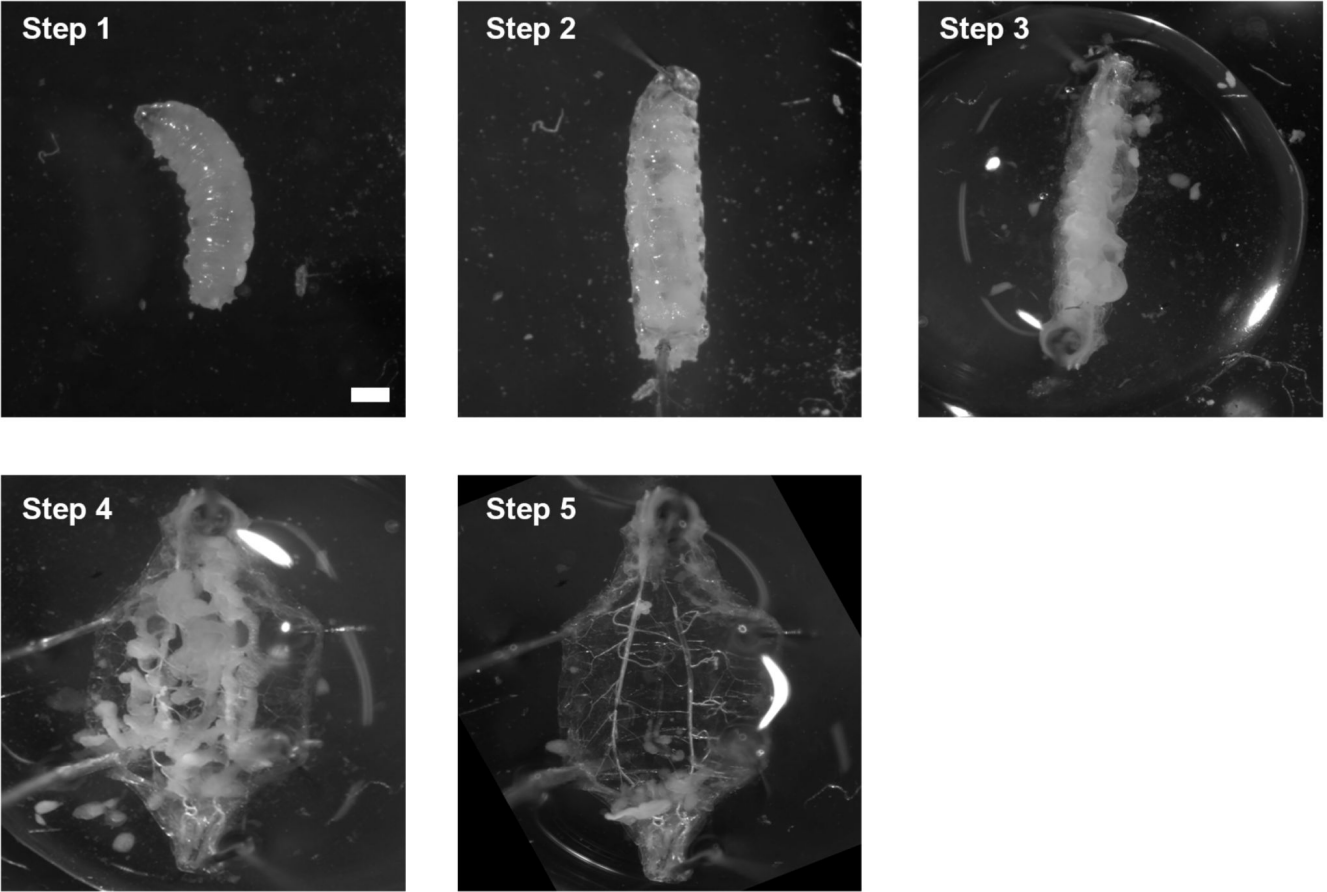
Dissection process of 3^rd^ instar larva Step 1: Put the larva on the Petri dish; Step 2: Use insect pins to secure the larva; Step 3: Open the body from bottom to top; Step 4: Secure the open cuticle with another 4 insect pins; Step 5: Remove the organs. Scale bar ≈ 0.25 mm.

1. Larvae are grown at 29 °C to the 3^rd^ instar stage. Six to ten control larvae (lacking RNAi transgene) are selected on the basis of GFP expression in the head, detected by fluorescence stereo microscopy (the GFP marker is linked to CyO on the inherited balancer chromosome). Six to ten experimental larvae carrying a UAS-RNAi transgene expressed in cardioblasts are identified by the absence of GFP marker expression (Brent *et al*., 2009).
2. Using forceps insert insect pins into tail and head to affix larvae to Petri dish, ventral side UP.
3. Submerge each larva under a drop of Schneider’s *Drosophila* medium.
4. Using scissors, make a ventral incision through the cuticle from tail to head.
5. Insert another 4 insect pins to hold open the excised cuticle.
6. The internal organs are carefully removed using No. 5 forceps (Figure 7).
7. Wash the remaining larva carcass 1 × with Schneider’s *Drosophila* medium.
8. The remaining steps are identical to the adult protocol.

## Data analysis

1. Use Freehand selection of ImageJ to carefully select the same area of all tissue samples.
2. Cardiac myofibrillar density, cardioblast cell numbers, and Pericardin deposition are quantified.
3. Use PAST.exe to perform statistical analysis. Sample error is presented as standard error of the mean (SEM). First test results for normality using the Shapiro-Wilk test (a = 0.05). Analyze normally distributed data by Student’s *t*-test (two groups) and Bonferroni comparison to adjust *P* value, or by a one-way analysis of variance followed by a Tukey-Kramer post-test for comparing multiple groups. Analyze non-normally distributed data by either a Mann-Whitney test (two groups) and Bonferroni comparison to adjust the *P* value, or a Kruskal-Wallis H-test followed by a Dunn’s test for comparisons between multiple groups. Statistical significance is defined as *P* < 0.05.

## Notes

1. All samples are imaged 1× only to avoid bleaching. All samples are imaged the same day at the same light intensity and exposure time.
2. No ventral muscle layer is present at 3^rd^ instar larva stage.
